# Clot Lysis and Antimitotic Study of *Ficus glomerata* Roxb Fruit Extracts

**DOI:** 10.1155/2014/975303

**Published:** 2014-03-31

**Authors:** Kirankumar Shivasharanappa, Ramesh Londonkar

**Affiliations:** Department of Biotechnology, Gulbarga University, Gulbarga, Karnataka 585106, India

## Abstract

The present study was carried out to investigate the thrombolytic and antimitotic potentiality of various extracts of fruits of *Ficus glomerata*, a traditional medicinal plant, using an *in vitro* assay method. Three crude extracts such as petroleum ether (FGPE), chloroform (FGCE), and methanol (FGME) were used for the study, with a standard (streptokinase) and negative control (sterile distilled water) to validate the method. The thrombolytic nature of the plant was found significant with methanol extract and chloroform and petroleum ether extracts have recorded mild activity, when compared with the negative control (sterile distilled water). The extracts have shown mild clot lysis, that is, 2.16%, 23.06%, 27.60%, and 47.74% of sterile distilled water, FGPE, FGCE, and FGME, respectively, while the standard (streptokinase) has shown 74.22% clot lysis. FGME inhibited the root growth in number as well as length effectively, followed by FGPE, while FGCE exhibited moderate antimitotic activity and it was supported by mitotic index. Therefore, the obtained results suggest that among all the extracts of plant the methanolic extract has shown highest thrombolytic and antimitotic activity.

## 1. Introduction

*Ficus glomerata* Roxb (Moraceae) is widely used in folk medicine for the treatment of various diseases [1]. The fig tree possesses antibacterial, antifungal, antiseptic, and analgesic qualities [2–5]. Thus, the present study is aimed at screening of different extracts of* Ficus glomerata* fruit for its clot lysis (thrombolytic activity) and antimitotic activity by using an* in vitro* assay method. These pharmacological activities have been selected because of their great medicinal relevance. In recent years, it is observed that the heart diseases are increasing to a great extent and side effects of synthetic drugs are becoming an ever-increasing therapeutic problem. Hence, it is needed to find out the safe, less or no side effective herbal drugs, because natural products of higher plants may give a new source of thrombolytic agents, as well as antimitotic agents [6].

A blood clot (thrombus) develops in the circulatory system due to failure of homeostasis which causes vascular blockage and while recovering leads to serious consequences in thrombotic diseases such as myocardial or cerebral problems, at times leading to death [7]. Thrombolytic agents that include tissue plasminogen activator (t-PA), urokinase (UK), and streptokinase (SK) are used across the world for the treatment of these diseases. In India, though streptokinase and urokinase are widely used due to lower cost, [8, 9] as compared to other thrombolytic drugs. But due to the weak substrate specificity of these first generation drugs (streptokinase and urokinase), they lead to systemic fibrinolysis, anaphylactic reaction, and bleeding complications (hemorrhage) [10]. Again, multiple treatments with streptokinase are restricted in a given patient as a result of immunogenicity [11]. Because of the shortcomings of the available thrombolytic drugs, attempts are underway to develop improved recombinant variants of these drugs [12, 13].

The antimitotic activity was screened using* Allium cepa* root meristematic cells which have been used extensively in screening of drugs with antimitotic activity [14, 15]. The roots of all plants have distinguished regions, one of them being the region of cell division that lies beyond the root cap and after this it even extends a few mm. Cells of this region undergo repeated divisions, and the fate of cell division is higher in this region compared to that of the other tissues; hence, this region is called the meristematic region [16]. This rapid division of meristematic cells is similar to that of the cancer cell division in humans. Hence, these meristematic cells can be used for preliminary screening of drugs with anticancer activity. Even though doubts can be raised about extrapolation of results from plant tissue to animals and finally to humans, plant cells are 1000 times more resistant to colchicines which are a potent anticarcinogen and act by inhibiting the microtubule formation. It is inferred that the chemicals which affect plant chromosomes will also affect animals [17].

## 2. Materials and Methods

### 2.1. Plant Material

Fresh ripe fruits were collected from Botanical Garden of Gulbarga University, Gulbarga. Fruits are washed, shade-dried, and ground into fine powder and kept in room temperature.

### 2.2. Chemicals

Streptokinase lyophilized (Solonase, 15,00,000 I.U. Cadila Health Care Limited) was purchased from local medical store, vincristine sulphate was purchased from Sigma Aldrich, and phosphate buffer saline, methanol, and all other chemicals were purchased from Merck pvt Ltd.

### 2.3. Extract Preparation

Powdered sample was subjected to extract in soxhlet extraction using petroleum ether, chloroform, and methanol solvents successively. Collected extract was centrifuged at 5,000 rpm for 10 min and filtered. The filtrate was used to conduct further study.

### 2.4. Blood Sample

2 mL of blood was drawn from tail veins of healthy albino rats, using a standard protocol approved by Institutional Ethical committee, Gulbarga University, Gulbarga. Collected blood was transferred to six Eppendorf tubes and allowed to form a clot.

### 2.5. *Allium cepa*

Healthy and fresh onion bulbs weighing 40–50 g were purchased from the local market of Gulbarga, Karnataka, India. Onions with green leaves/shooting, moldy, and dried were discarded, whereas healthy, disease free onion bulbs were taken for the study.

### 2.6. *In Vitro* Clot Lysis Activity

Clot lysis activity was carried out using the method of Prasad et al., 2006 [18]. Venous blood drawn from the healthy albino rats was distributed in five different preweighed sterile microcentrifuge tubes and incubated at 37°C for 45 min. After clot formation, serum was completely removed without disturbing the clot and each tube having clot was again weighed to determine the clot weight (clot weight = weight of clot containing tube − weight of tube alone). To each microcentrifuge tube containing preweighed clot, 100 *μ*L of plant extracts was added separately. As a positive control, 100 *μ*L of streptokinase and, as a negative nonthrombolytic control, 100 *μ*L of sterile distilled water were separately added to the control tubes. All the tubes were then incubated at 37°C for 90 min and observed for clot lysis. After incubation, fluid released was removed and tubes were again weighed to observe the differences in weight after the clot disruption. Difference in weight was taken before and after the clot lysis and it was expressed as percentage of clot lysis. Percentage of clot lysis was calculated using the formula:

(1) Percentage  of  Clot  lysis=(weight  of  released  clotclot  weight) ×100.

### 2.7. Antimitotic Assay in Onion Root Tips

Antimitotic activity of* Ficus glomerata* fruit extracts was carried out by using the method described by Fiskesjo, 1993 [19]. Dried outer layers of healthy onion bulbs were removed and placed over a series of jars containing normal tap water, until to grow 3-4 cm of roots from each bulb and tap water was changed at interval of 24 hrs. After the root development, bulbs were considered as viable bulbs and water content was removed using tissue paper and these bulbs were selected for the study. These roots were treated with petroleum ether, chloroform, and methanol extracts whereas the positive control is treated with vincristine sulphate. A blank with tap water was used as negative control. After 72 hr of treatment, the roots were taken out and total root length and number of roots per bulb were measured and root tips were cut and transferred to fixing solution acetic acid: ethanol in the ratio of 1 : 3 and slightly warmed in 1 N HCl. The treated root tips were then stained with acetocarmine. The slide was observed under microscope to count the number of cells, nondividing, and dividing cells. Mitotic index was calculated using the following formula:

(2) $$\begin{array}{c} \text{Mitotic}\;\text{Index}=\left(\frac{\text{Number}\;\text{of}\;\text{dividing}\;\text{cells}}{\text{Total}\;\text{number}\;\text{of}\;\text{cells}}\right)\times 100. \end{array}$$

## 3. Results

### 3.1. Clot Lysis

As a part of discovery of cardioprotective drugs from natural resources, the different extractives of* Ficus glomerata* were assessed for thrombolytic activity. In this test, addition of 100 *μ*L SK (streptokinase), a positive control (30,000 I.U.), to the clots and subsequent incubation for 90 minutes at 37°C have shown 74.65 ± 3.606% lysis of clot. On the other hand, sterile distilled water was treated as negative control which exhibited negligible percentages of lysis of clot (4.50 ± 1.110%). The mean difference in clot lysis percentage between positive and negative control was found statistically very significant. The* in vitro* thrombolytic activity study revealed that methanol extract of* Ficus glomerata* fruits exhibited highest thrombolytic activity (47.23 ± 2.778%). However, significant thrombolytic activity was demonstrated by the petroleum ether and chloroform extracts which have shown 27.89 ± 3.418% and 23.11% ± 2.656%, respectively.

**Figure 1:**
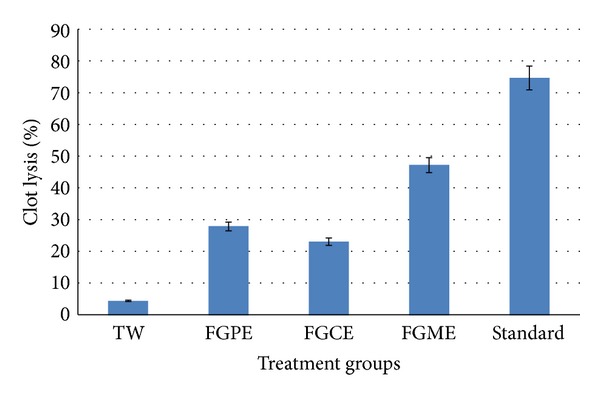
Clot Lysis activity of* Ficus glomerata* fruit extracts (FGCE:* Ficus glomerata* chloroform extract, FGME:* Ficus glomerata* methanol extract, FGAE:* Ficus glomerata* aqueous extract, and standard: streptokinase).

Graphical representation of the effective clot lysis percentage by various solvent extracts of* Ficus glomerata* fruits, positive thrombolytic control (Streptokinase), and negative control (sterile distilled water) is given in Figure 1.

### 3.2. Antimitotic Activity

**Figure 2:**
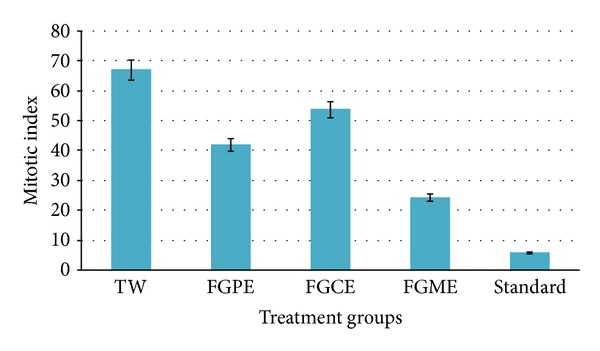
Antimitotic activity of* Ficus glomerata* fruit extracts (TW: tap water, FGCE:* Ficus glomerata* chloroform extract, FGME:* Ficus glomerata* methanol extract, FGAE:* Ficus glomerata* aqueous extract, and standard: streptokinase).

The results obtained are summarized (Table 1 and Figure 2). It is observed that* Ficus glomerata* fruit extracts stunted the growth and development of onion roots. In addition, the number of roots and average root length and mitotic index were lesser in that plant which is exposed to fruit extracts than control groups. Out of three extracts, methanol extract was found highly effective in reduction of root number, root length, and mitotic index followed by chloroform extract and petroleum ether extract as compared to normal control group which is treated with tap water.

## 4. Discussion

The results in the present study showed that the extracts of* Ficus glomerata* have mild to moderate antimitotic and clot lysis activity. The significant average percent of clot lysis is observed due to methanol extract (47.23%), moderate by chloroform extract (27.89%), and least by petroleum ether extract (23.11%) treatment, whereas the standard thrombolytic drug streptokinase treatment has showed 74.65% of clot lysis. On the other hand methanolic extract has stopped the root growth significantly, followed by petroleum ether extract and chloroform extract. These antimitotic activities were supported by mitotic index. This effect may be possibly due to phytoconstituents present in the plant extracts affecting the cytoskeleton or inhibiting the activity of one or more components of the cell cycle, thereby providing clear information regarding cytotoxic action of fruit extracts as reported by Bhattacharya and Haldar 2010 [20]. Growth inhibition effect may be due to diminished cell division [21]. Therefore, it is evident that the plant extracts possess antimitotic activity.

Onion roots have been used as a model for the study of phytotoxic activity as per Williams and Omoh (1996) who studied other similar systems [22].* Allium cepa* has been used for evaluating cytotoxicity since the early 1920s [23]. This method is an easy and sensitive tool for measuring the total toxicity caused by chemical treatments as expressed by growth inhibition of the roots of onion bulbs. Levan (1938) [24] has reported that* Allium* test is a rapid, highly sensitive, and reproducible bioassay for detecting cytotoxicity of phytochemicals and results of* Allium* test fit well for prokaryotes and other eukaryotes.

Clot lysis may be the result of the combinatorial effect of the active compounds present or the individual compounds [25]. With further research on cell viability tests and* in vivo* studies, this finding may have important implications in the treatment of cardiovascular diseases, which is increasing at an alarming rate. Since the drugs used for the cardiovascular diseases are not economical and not accessible to the greater section of the society and they cause side effects, herbal drugs are having less or no side effects and application of phytomedicine may be a boon for the above diseased patients [26].

## 5. Conclusion

From this study, it could be suggested that* F. glomerata* is a promising source of natural drug which has the ability to modify the physiological function of cells and hence acts as anticancer drugs to arrest the proliferation of cancer cells. Therefore, it can be concluded that the* Ficus glomerata* extracts have shown a commendable antithrombotic activity.

## Figures and Tables

**Table 1:** Effect of *Ficus glomerata* extracts on roots of *Allium cepa*.

Plant extracts/control	Number of roots	Average root length (in cm)
TW	13	4.31 ± 0.23
FGPE	8	3.58 ± 0.47
FGCE	11	4.17 ± 0.13
FGME	7	3.11 ± 0.56
Standard	5	2.19 ± 0.27

TW: tap water, FGCE:* Ficus glomerata *chloroform extract, FGME:* Ficus glomerata* methanol extract, FGAE: *Ficus glomerata* aqueous extract, and standard: streptokinase.
